# Variation in Seed Allergen Content From Three Varieties of Soybean Cultivated in Nine Different Locations in Iowa, Illinois, and Indiana

**DOI:** 10.3389/fpls.2018.01025

**Published:** 2018-07-23

**Authors:** Scott McClain, Severin E. Stevenson, Cavell Brownie, Corinne Herouet-Guicheney, Rod A. Herman, Gregory S. Ladics, Laura Privalle, Jason M. Ward, Nancy Doerrer, Jay J. Thelen

**Affiliations:** ^1^Syngenta Crop Protection, LLC, Research Triangle Park, NC, United States; ^2^Department of Biochemistry, Christopher S. Bond Life Sciences Center, University of Missouri, Columbia, MO, United States; ^3^Department of Statistics, North Carolina State University, Raleigh, NC, United States; ^4^Regulatory Science, Bayer SAS, Sophia Antipolis, France; ^5^Dow AgroSciences, Zionsville, Indianapolis, IN, United States; ^6^Pioneer Hi-Bred, DuPont Agricultural Biotechnology, Wilmington, DE, United States; ^7^BASF Plant Science, Research Triangle Park, NC, United States; ^8^Regulatory Affairs, Royal Canin, U.S.A., St. Charles, MO, United States; ^9^Protein Allergenicity Technical Committee, Health and Environmental Sciences Institute, Washington, DC, United States

**Keywords:** allergen, soybean, quantitative proteomics, mass spectrometry, multiple reaction monitoring, AQUA

## Abstract

Soybean (*Glycine max*) is an important food stock, and also considered an allergenic food with at least eight well characterized allergens. However, it is a less prevalent allergen source than many other foods and is rarely life-threatening. Soybean is incorporated into commonly consumed foods, and therefore, the allergens pose a potential concern for individuals already sensitized. The protein profile of soybean can be affected by several factors including genetic and environmental. To investigate how soybean allergen content may be affected by genetics and/or environment, nine soy allergens were quantified from three commercial soybean varieties grown at nine locations in three states within a single climate zone in North America; Iowa, Illinois, and Indiana, United States. Quantitation was achieved using liquid chromatography-selected reaction monitoring (LC-SRM) tandem mass spectrometry with AQUA peptide standards specific to the nine target allergens. Quantitation of allergen concentration indicated that both genetics and location affected specific allergen content. Seven of the nine allergens were significantly influenced by genetics, with the exceptions of glycinin G4 and KTI 3. The allergens P34, Gly m Bd 28k, glycinin G3, and KTI 1 showed statistically significant impact from location as well, but at a lower threshold of significance compared with genetics (cultivar/variety). This dataset contributes to our understanding of the natural variation of endogenous allergens, as it represents a sampling of soybeans grown in a controlled, distributed plot design under agronomic conditions common for commercial soybean food and feed production. The aim was to build upon our recent understanding of how allergens are expressed as part of the overall soybean proteome.

## Introduction

Soybean is used in the manufacturing of a vast variety of foods, but is considered a major allergenic food in the United States, and much of the developed world. Over 30 potential soybean allergen sequences have been identified, sixteen of which have been confirmed with some data to support sensitization and elicitation (Stevenson et al., 2012). A comprehensive listing of peer-reviewed allergens is available^1^ with a recent review finding that eight soybean allergens have a clear status as both clinically relevant allergens with known sequences (Ladics et al., 2014). Other databases or listings of allergens are also available such as www.allergenonline.org and www.allergome.org. The primary soybean allergens are seed storage proteins which are of particular interest in certain foods because of their physical and nutritional properties. These properties include gelation, pH, solubility as well as emulsification; they are known to affect the way foods are processed (Utsumi and Kinsella, 1985; Cai and Chang, 1999; Mujoo et al., 2003). Although soybean is a widely recognized allergenic food crop, a specific and multiplexed method to quantitatively measure protein allergens has only recently been developed and utilized (Houston et al., 2011; Stevenson et al., 2012).

One expectation of any newly developed analytical capability that is tied to human health is the evaluation of important variables affecting the interpretation of quantitative expression data, particularly in the case of allergens where determining exposure thresholds can be critical. As has been noted by initial work with soybeans using tandem mass spectrometry approaches, absolute quantitative analysis of allergen expression in food is novel (Houston et al., 2011; Stevenson et al., 2012). Analytically robust studies are essential to provide useful information on the natural variability of allergens. In the case of soybean, understanding the genetic variation in soybean and the impact from environmental conditions on allergen content is important because: (1) there are many individual allergens and the relationship of expression between one or more of the allergens has not been fully characterized; (2) soybeans are planted across broad geographical locations and environmental influence is known to be a primary variable in allergome profiles (Stevenson et al., 2012); and (3) soybeans have a wide array of uses that rely on extensive breeding which creates a variety of genome profiles capable of potentially impacting the expressed proteome. However, commodity soybeans are blended from numerous field sources and individual varieties are not generally considered a point-source of food exposure. Therefore, slight differences in allergen content on a variety basis might be inconsequential in the context of allergen exposure from commodity soybeans and food products. Nevertheless, an understanding of the natural variability of seed allergen content is an expectation on a variety of fronts, including regulatory questions around genetically modified soybean varieties and in commodity soybean processing, as soybean is likely to remain a prominent food allergen source on a population and allergy prevalence basis. From a technical standpoint, describing the variance of target analytes (the soybean allergens in this case) is a key to using any assay for describing significant variance under controlled experimentation. This is particularly true in understanding a complex plant response to factors that may affect how much allergen load an allergen source carries.

In this study, nine allergens (or sub-units of multimers) that have each been substantially characterized with respect to their allergenicity were chosen for analysis (Utsumi, 1992; Stevenson et al., 2012) with two of the Kunitz trypsin inhibitors (KTI 1 and KTI 3) included. In some cases, these allergens exist *in vivo* as heteromers, necessitating the measurement of individual subunits during quantitation. For example, glycinin accounts for at least 30% of the soybean seed protein and consists of six subunits; each subunit has a specific basic chain linked to an acidic chain via disulfide bonding. The assembly of the basic and acidic subunits is achieved through a series of co- and post-translational modifications of the original polypeptides known as glycinin precursors (Ereken-Tumer et al., 1981; Utsumi, 1992; Petruccelli and Anon, 1995; Riblett et al., 2001). We were able to determine the absolute quantities (i.e., concentrations) of one chain from each of the four glycinin (11S globulin; Gly m 6) subunits (glycinin G1, Bx chain; glycinin G2, A2 chain; glycinin G3, A chain; glycinin G4, A5 chain). The second most abundant storage protein in soybean, β-conglycinin (vicilin; Gly m 5), a trimer, consists of α′, α, and β subunits (Mori et al., 1981) and the available protein sequence for the α-subunit of β-conglycinin was used in this study. Both Gly m Bd 30K (P34) and Gly m Bd 28K allergens are Asn-linked glycoproteins belonging to the thiol protease family (Ogawa et al., 1993; Tsuji et al., 1997). The P34 protein is known to elicit severe allergic reactions even at low abundance and is one of the most immune-dominant soybean allergens (Herman, 2003).

The goal of this work was to investigate and compare two variables that could quantitatively influence soybean seed allergen content; geographical growth location (environment; external influence) and genetics of the soybean varieties (genotype; internal influence). To accomplish this, samples were taken of soybeans from three varieties grown together at nine different field locations, but within a single climate zone representing the major soybean growing region in the United States. Although within this one growing zone, it was expected that environmental conditions vary from site to site since locations were spread apart through three states of United States. The goal of utilizing samples of soybean from this field trial across many sites was to examine the hypothesis that the combination of broadly assumed environmental variance and genetics of the soybean varieties would offer insight into how protein expression varies for important soybean allergens. It is recognized that although some characterization of environmental conditions are recorded for the underlying field trial from which samples were obtained, the work herein is a parallel examination of specific protein allergen expression that is apart from the field trial design itself since quantitative measures of environmental parameters were not included in the allergen statistical analysis and were considered beyond the scope of this allergen expression survey.

Nine allergens were quantitatively measured to provide a basis for interpreting the most influential variable on the expression of each allergen. These sequences were previously available as part of the FARRP allergenonline.org database for which there was pertinent sequence information and high quality clinical data supporting these as relevant allergens. A recent study that compared allergen levels of four non-GM soybean varieties grown in three different climate zones in North America showed that environment had a greater impact on allergen expression variability than genetics (Stevenson et al., 2012). In this paper, we address the same interacting variables by comparing three soy varieties grown at nine locations within a single North American climate zone. It is understood that each soybean variety has inherent, but undefined differences in background genetics that likely influence the final compositional makeup of soybean seed, including protein allergens. The environmental “load” or putative impact from the environment is characterized by a selection of varieties adapted to single climate zone having been grown at nine distinct locations that have differences in seasonal agronomic conditions.

## Materials and Methods

### Plant Material

Three soybean varieties, A, B1, and B2, grown in 2009 in nine replicated field trials were used for analysis. The varieties are branded soybean seed produced in bulk as commodity soybean fit for growing in the United States region denoted in this study. They are listed and then coded with the letter designation for discussion herein as the following: A = Stine 2500-2 Brand; B1 = Stine 3300-2 Brand; B2 = Stine 3308-2 Brand. All three are non-transgenic and commercially available varieties and represent two different maturity groups; A is maturity group 2; B1 and B2 are group 3. Each field trial consisted of single plots of each variety and all plots at a site were subjected to the same agronomic practices during the 2009 United States growing season. Samples of 20 g each were taken from the harvest of one plot for each variety at each location. The field trials were located in three contiguous mid-western states in the United States: Iowa (IA), Illinois (IL), and Indiana (IN) (Supplementary Figure 1). IA was the location of seven of the nine plots, near the towns of Adel, Glidden, IA Falls, Marcus, Mediapolis, Perry and Winterset, and one plot each was located close to Fithian, IL, and Sharpsville, IN. Site-specific soil description and weather conditions are described in Supplementary Table 5.

### Protein Extraction and Quantification

Total protein was extracted from three sub-samples (five seeds/sub-sample) of soybean seed taken from each variety at each site. Each sub-sample was ground to a fine powder using a Waring blender. A phenol extraction method compatible with soybean was employed similarly to Hajduch et al., 2007. Briefly, ground soybean material was suspended in 2.5 mL of Tris-buffered phenol (pH 8.0; Fisher Scientific, Inc., Pittsburgh, PA, United States) and 2.5 mL extraction buffer [0.9 M sucrose, 0.1 M Tris–HCl pH 8.0, 10 mM EDTA, 0.4% (v/v) 2-mercaptoethanol]. The mixture was homogenized with a T25 Basic S1 Disperser (IKA Works, Inc., Wilmington, NC, United States) on speed 4 with the S25N-10 tool for 2 min. The homogenized mixture was inverted at 4°C for 2 h and then centrifuged at 5000 × *g* and 4°C for 20 min. The phenol phase was removed, and a single back extraction was performed using an additional 2.5 mL of phenol following the same procedure. Phenol fractions were combined, and protein precipitated using five volumes of 95% (v/v) methanol with 0.1 M ammonium acetate then incubating at -20°C overnight. The precipitated protein was pelleted by centrifugation at 5000 × *g* and 4°C for 5 min and washed by re-suspending in 10 mL fresh methanol-ammonium acetate solution three times, 80% (v/v) acetone two times, and 70% (v/v) ethanol once, with 20 min incubations at -20°C before pelleting for each. Protein was stored in 80% (v/v) acetone at -20°C. The amount of total protein extracted was quantified as in Stevenson et al. (2012) by using the Bradford (1976) method.

### In-Solution Digestion and Mass Spectrometry Analysis

Protein was aliquoted and digested prior to mass spectrometry as described in Lee et al. (2010) and Stevenson et al. (2012). Briefly, frozen previously quantitated protein was thawed at 4°C, vortexed until homogenous and 10 μg portioned into 1.5 mL polypropylene tubes. Sample volumes equalized by bringing to 10.0 μL using 8 M urea buffer. Disulfide bonds were reduced with dithiothreitol (DTT), to 10 mM for 1 h at room temperature. Reduced cysteines were carboxyamidomethylated with iodoacetamide to 50 mM, at room temperature in the dark for 1 h. Urea was diluted to 0.67 M and iodoacetamide was neutralized with equimolar DTT and incubating at room temperature for 15 min. Samples were chilled to 4°C and trypsin (sequencing grade – modified, Promega, Madison, WI, United States) added to 1.7 ng/mL. Digestion was allowed to proceed for 16 h at 37°C. After digestion, reactions were cooled to 4°C and each preparation was spiked with AQUA peptide standards (Sigma-Aldrich, The Woodlands, TX, United States) in 50% acetonitrile (Sigma-Aldrich, Saint Louis, MO, United States) and 1% (v/v) formic acid (ACS grade, 88% purity; Acros Organics, NJ, United States) within their linear quantitative ranges, determined as described by Houston et al. (2011). Typically, this is 100 fmol of peptide standard per 1.0 μg of soy protein, targeting each standard within a 10-fold concentration range of the native peptide signal. Target peptide and protein surrogate information are displayed in Supplementary Table 1. Digests containing peptide standards (for each allergen) were frozen at -80°C and lyophilized to dryness using a Centrivap. Lyophilized digests were dissolved to 0.2 μg/μL in 5% (v/v) acetonitrile, 0.1% (v/v) formic acid by vortexing and were centrifuged at 21,000 × *g* for 2 min at room temperature. The supernatant was transferred to a 96-well plate for LC-SRM analysis.

Mass spectrometric analysis was performed similarly to the previously described method in Stevenson et al. (2012), with 1 μg on column (5 μL loadings) using a TSQ Vantage EMR (Thermo Fisher Scientific Inc., San Jose, CA, United States), which was within the linear range for quantification for all peptides. Automated sampling and chromatography were performed using an Eksigent nanoLC-Ultra 1D plus (AB SCIEX, Dublin, CA, United States). Sample peptides were desalted and chromatographically separated using reverse-phase chromatography with acidified acetonitrile and water mobile phases. Separations were achieved using a 25-min run with a 12.5-min non-linear gradient from 2 to 60% acetonitrile at 500 nL/min. Peptides were measured as they eluted in pre-determined retention time windows, with dwell times optimized to obtain at least ten measurements across the chromatographic peak with at least five transitions monitored per peptide (SRM details provided in Supplementary Table 2). All chromatographic elution profiles for peptides of interest were scrutinized to ensure matching retention times and relative fragment ion intensity ratios (for AQUA and native peptide precursor and product masses, respectively). Peak integration and calculations were performed by LCQuan software (Thermo Fisher Scientific Inc., San Jose, CA, United States). Absolute quantities were calculated by multiplying the response ratio (sum of the fragment mass peak areas for endogenous peptide signal/sum of the fragment mass peak areas for AQUA peptide signal) by the amount of AQUA peptide analyzed.

### Statistical Analysis

These analyses were based on the following linear model for the concentrations (μg/mg) for a single allergen:

(1) $$\begin{array}{c} Y_{ijk}=\mu +G_{i}+L_{j}+GL_{ij}+\varepsilon_{ijk} \end{array}$$

where Y _ijk_ is the concentration of protein for the *k*th sample from Variety *i* in Location *j, μ* is the overall mean, *G_i_* is an effect for the *i*th Variety, *L_j_* is an effect for the *j*th Location, *GL_ij_* is a Variety by Location interaction effect, and 𝜀_ijk_ is a random error that includes sampling error and measurement error.

The SAS ^2^ (SAS Institute, Inc., Cary, NC, United States) procedure for general linear models (Proc GLM) was used to carry out the mixed effects ANOVA based on the linear model above with variety defined as a fixed factor. The analysis was carried out separately for each allergen. Both transformed and untransformed raw data were used to initially survey the data analysis. Resulting *F*-tests for analysis of untransformed data are summarized in **Table 1**.

**Table 1:** *F*-tests for significance: genotype (variety), location, and variety by location interaction effects.

Allergenic protein	Numerator source	Denominator mean square source	Num DF^∗^	Den DF^∗∗^	*F*-value	*p*-value
P34 (Gly m Bd 30k)	Genotype	Genotype × Location	2	16	19.60	0.000
P34 (Gly m Bd 30k)	Location	Genotype × Location	8	16	5.60	0.002
P34 (Gly m Bd 30k)	Genotype × Location	Residual	16	54	9.42	0.000
β-conglycinin α-subunit	Genotype	Genotype × Location	2	16	250.17	0.000
β-conglycinin α-subunit	Location	Genotype × Location	8	16	1.60	0.202
β-conglycinin α-subunit	Genotype × Location	Residual	16	54	1.94	0.036
Gly m Bd 28k	Genotype	Genotype × Location	2	16	8.71	0.003
Gly m Bd 28k	Location	Genotype × Location	8	16	3.56	0.015
Gly m Bd 28k	Genotype × Location	Residual	16	54	3.03	0.001
Glycinin G1	Genotype	Genotype × Location	2	16	74.19	0.000
Glycinin G1	Location	Genotype × Location	8	16	1.58	0.208
Glycinin G1	Genotype × Location	Residual	16	54	2.43	0.008
Glycinin G2	Genotype	Genotype × Location	2	16	26.56	0.000
Glycinin G2	Location	Genotype × Location	8	16	1.47	0.242
Glycinin G2	Genotype × Location	Residual	16	54	2.91	0.002
Glycinin G3	Genotype	Genotype × Location	2	16	6.73	0.008
Glycinin G3	Location	Genotype × Location	8	16	6.57	0.001
Glycinin G3	Genotype × Location	Residual	16	54	33.78	0.000
Glycinin G4	Genotype	Genotype × Location	2	16	1.76	0.204
Glycinin G4	Location	Genotype × Location	8	16	1.62	0.197
Glycinin G4	Genotype × Location	Residual	16	54	3.35	0.000
KTI 1	Genotype	Genotype × Location	2	16	276.81	0.000
KTI 1	Location	Genotype × Location	8	16	3.17	0.024
KTI 1	Genotype × Location	Residual	16	54	10.10	0.000
KTI 3	Genotype	Genotype × Location	2	16	1.42	0.270
KTI 3	Location	Genotype × Location	8	16	1.24	0.339
KTI 3	Genotype × Location	Residual	16	54	6.55	0.000
β-conglycinin α-subunit/total Glycinin	Genotype	Genotype × Location	2	16	10.12	0.001
β-conglycinin α-subunit/total Glycinin	Location	Genotype × Location	8	16	3.20	0.074
β-conglycinin α-subunit/total Glycinin	Genotype × Location	Residual	16	54	32.67	0.000

*The source of the numerator in this calculation was varied between genotype, location, and genotype^∗^location. The source of the Mean Square used in the denominator was either genotype^∗^location or residual. ^∗^Num DF = numerator (as indicated in the Source column) degrees of freedom. ^∗∗^Den DF = denominator (as indicated in Denominator Mean Square column) degrees of freedom.*

The ratio of β-conglycinin α-subunit to total glycinin concentration was calculated for each sample, and ANOVA was carried out on the ratios to test whether the ratio differed by variety or location. Results for *F*-tests are at the bottom of **Table 1**.

Multivariate analysis (MANOVA) was used to demonstrate the presence of variety-by-allergen interaction for the summed allergen groups, β-conglycinin α-subunit, total glycinin, and total KTI. Pairs of each of these allergen groups were analyzed to evaluate variety (genotype) and location effects and the possible interaction of these variables with variation of the allergen pairs (see Supplementary Table 3 for analysis and *p*-values). MANOVA was performed on the summed concentration values for the four glycinin sub-units (total glycinin group) and the summed values for the two KTI sub-units (total KTI); log concentration values were used for analysis. The allergens P34 and Gly m Bd28k were the least abundant allergens and were excluded from MANOVA analysis.

## Results

### Influence of Environment and Genetic Background on Allergen Expression

**FIGURE 1:**
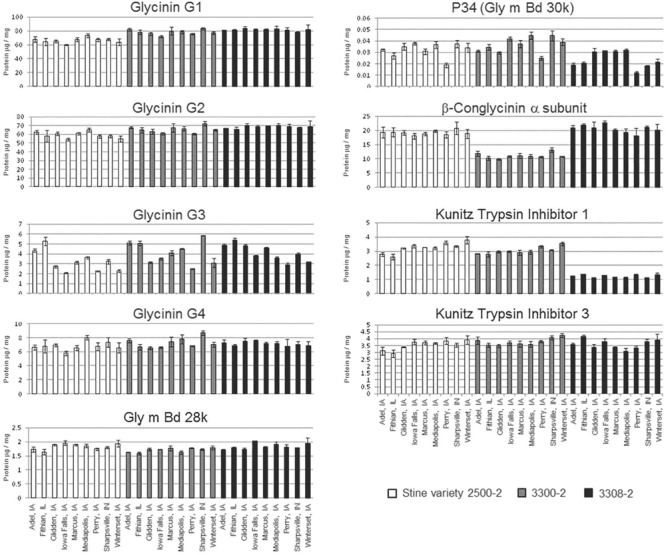
Concentration of nine allergens by location and soybean variety. The concentration of each allergen (μg/mg total protein) is plotted separately in each panel for each allergen. The grayscale shading differentiates the three varieties and the *X*-axis provides the growth location. Error bars denote standard deviation from three replicates (sub-samples of grain, each prepared separately). Statistical analysis of this data is presented in **Table 1**.

Three varieties of soybean were grown and harvested from nine locations in a single climate zone in North America (Supplementary Figure 1). There were significant differences for each of the allergen concentrations [except for glycinin G4 (Gly G4) and Kunitz trypsin inhibitor (KTI 3)] across each sample type, due to either location or soybean variety (**Figure 1**). Variety and location effects (including statistical interaction) explained a significant fraction of the statistical variation for all allergens (**Table 1**). Soybean variety (genotype) showed the strongest effect statistically as all but glycinin G4 (Gly G4) and KTI 3 showed differences at a significance level of *p* < 0.01. And, of these seven showing strong impact from location, five of them had *p*-values past the third decimal (reported as < 0.000). In comparison, only four of the allergens, P34, Gly m Bd28k, glycinin G3, and KTI 1, showed significant influence from location and this was at a significance level of *p* < 0.05, but not nearly as consistently low in *p*-value as shown by that of variety. Variety effects were particularly strong (> 60% of total) for β-conglycinin α-subunit, glycinins G1 and G2, KTI 1, and also for P34 (about 40% of total).

As an example of the greater expression range (i.e., from smallest to largest concentrations) for KTI 1 and the easily distinguishable variety difference, the B2 variety had ∼3-fold lower concentration of KTI 1 compared to the other two varieties. Overall, KTI 1 and KTI 3 were detected at similar levels across site locations, 1.2–3.8 and 2.9–4.2 μg allergen/mg of protein, respectively, and were consistent with the published abundance data for these proteins (Stevenson et al., 2012). However, abundance of the KTI 1 allergen was affected differently compared to KTI 3; the KTI 1 concentration was significantly affected by the variety and by location, whereas KTI 3 was not significantly affected by either.

A statistical evaluation of the location effects showed that the strongest impact was on glycinin G3 and P34; about 50 and 30% of the total statistical variation, respectively (**Table 2**). Results are similar whether or not data were log-transformed (log-transformed data analyses not shown). Residual (i.e., within location and variety) variance was greatest for glycinin G4 and KTI 3, about 47 and 32%, respectively. Importantly, the residual variance displayed in **Table 2** helps to explain why neither glycinin G4 nor KTI 3 showed an impact from either variety or location as separate variables (**Table 1**); these are the two highest residual values indicating that some other variable must be explaining how these allergens are expressed. Note that these are the only two allergens with both location and variety demonstrating a non-significant impact on expression levels (*p*-values > 0.05; **Table 1**). The residual statistic is also highly significant (**Table 1**) for both of these allergens. Therefore, it is assumed that any variability in expression for glycinin G4 and KTI 3 allergens is largely a representation of sampling/experimental error or other variable not included in our analysis; it appears that neither environment nor variety has an impact on the levels of these two allergens.

**Table 2:** Relative contribution to total statistical variance of variety and location for each of the nine allergen concentrations: main and interaction effects.

		% of Total Variance Contributed by each Variable			
Allergenic protein	Total variance	Variety	Location	Variety by Location	Residual
P34 (Gly m Bd 30k)	8.89E-05	42.95	31.86	18.57	6.62
β-conglycinin α-subunit	28.23	92.55	0.67	1.62	5.16
Gly m Bd 28k	0.016	25.41	25.31	19.86	29.41
Glycinin G1	77.69	80.13	1.90	5.79	12.18
Glycinin G2	37.00	60.62	3.36	14.01	22.01
Glycinin G3	1.27	17.91	52.28	27.31	2.50
Glycinin G4	0.49	4.48	10.90	37.19	47.43
KTI 1	1.27	94.10	2.22	2.77	0.91
KTI 3	0.12	3.28	5.55	59.18	31.98

### Total Allergen Content of Three Commercial Varieties Grown in Nine Different Locations

**FIGURE 2:**
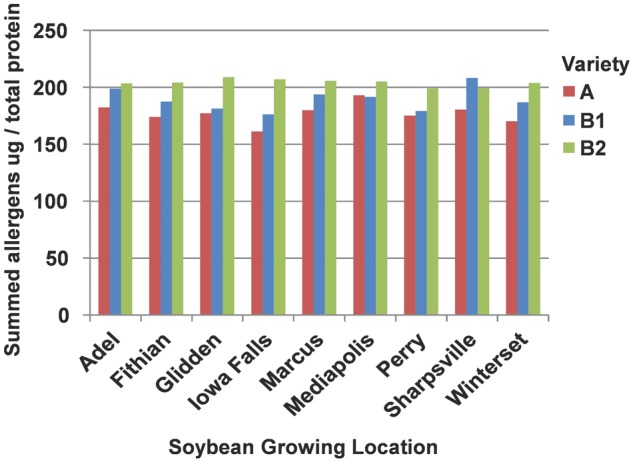
Summed allergen content by location for each soybean variety. The concentrations of each of the allergens were summed by location and plotted for each of the three soybean varieties.

The total allergen content for soybean sample was computed by summing the concentration values for all nine allergens (**Figure 2**); a breakdown of each allergen concentration as summed for the nine site locations and displayed for each variety is shown in Supplementary Table 4. The summed values for the allergens (**Figure 2**) are shown in order to represent the “total allergen load.” However, each allergen has independent distribution around the mean and summing the values across the allergens does not allow a convenient way to represent statistical spread around the mean. Therefore, the data bars are free of standard error or deviation bars. The lowest concentration of allergens was observed in variety A (149 μg/mg at IA Falls) and the highest level in variety B2 (193 μg/mg at Glidden). Variety B2 displayed the highest concentration at each location, except at Sharpsville (B1 was highest at 192 μg/mg for Sharpsville), and varied the least across all of the sites displaying a concentration differential from lowest to highest of approximately 9 μg/mg. In contrast, more variation from location to location was observed for the other two soybean varieties. The concentration differential (difference from lowest to highest value across locations) for variety A was approximately 29 μg/mg and the differential for the variety B1 was 30 μg/mg, both of which were more than three times greater in their differential (i.e., variation across locations) than that of the variety B2.

### Correlation Among Soybean Allergens

One question that was asked *a priori* with regard to the relationship of allergen content was whether the allergens correlated with other allergens according to their protein family. This analysis was approached by summing the concentration values for the allergen isoforms that group into a single protein or protein group; for example, glycinin and Kunitz trypsin inhibitor, respectively. The focus was limited to the most abundant of the allergen groupings in this study; the glycinin and Kunitz trypsin inhibitor allergens. The summed allergen types were then compared to the single representative of the conglycinin protein family, β-conglycinin α-subunit, also one the abundant allergens.

**FIGURE 3:**
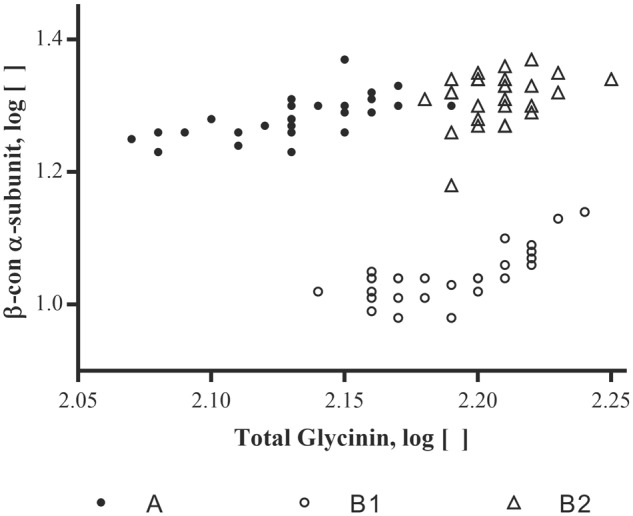
Soybean varieties plotted for their log concentrations of β-conglycinin α-subunit versus the log concentrations of the summed total of the glycinin subunits (Total glycinin). Log concentrations are of the mean μg allergen(s)/mg total protein; each site location per variety and allergen (*n* = 3).

To investigate possible correlations between changes in one allergen concentration versus that of another, log concentrations for one allergen were plotted against another for various pairs of allergens. β-conglycinin α-subunit was then plotted against each of these totals; an example plot is shown in **Figure 3**. Clustering of points associated with the three varieties is evident. This clustering suggests that variety impacts the relationships between allergens and thus, computing simple correlations between pairs of allergens, using all observations and ignoring variety, was not useful. MANOVA was therefore used to demonstrate the presence of variety-by-allergen interaction for the allergen pairs involving β-conglycinin α-subunit, total glycinin and total KTI.

**FIGURE 4:**
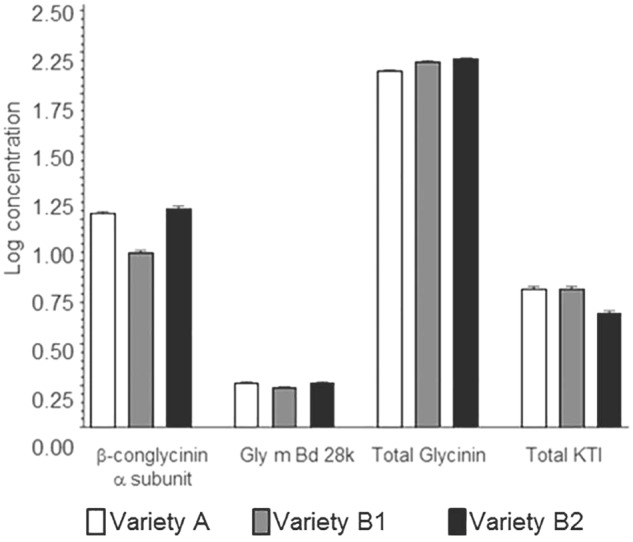
Mean log concentrations of protein allergens by group and variety. Paralogous protein allergen concentrations were summed (individual allergens summed together into allergen groups for glycinin and Kunitz trypsin inhibitor proteins, respectively, for each site location). Data displayed is the mean and standard deviation for each of the varieties at all site locations.

**FIGURE 5:**
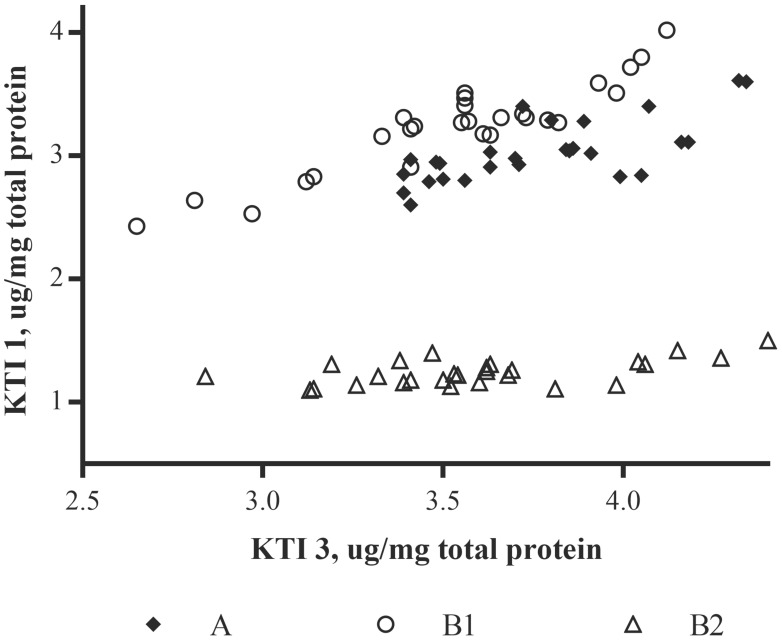
Plot of Kunitz trypsin inhibitor concentrations in soybean seed as measured by LC-SRM mass spectrometry; relationship between KTI 1 and KTI 3 concentrations relative to each of the three soybean varieties. Data represented is for each site location per variety and allergen (*n* = 3).

In applying additional MANOVA analyses (Supplementary Table 3), results showed that, as with single allergens or related allergen types in the prior analysis, the soybean variety (noted as genotype) had a significant impact on the relationship between allergens. As an example, when β-conglycinin α-subunit, total glycinin, and total KTI were each compared to the other allergens the variety was a significant variable; in most cases, growing location did not have a statistical impact unless the raw data was log-transformed (Supplementary Table 3; e.g., Total glycinin and total KTI; location *F*-value 2.73; *p*-value = 0.0133). With regard to the initial premise, the relationship between allergens was followed across the three varieties and demonstrated that an increase in one allergen type was shown to not necessarily be accompanied by a decrease in another (**Figure 4**; P34 data not shown due to the extremely low concentration values). This indicates that allergen expression does not vary in a predictable manner based on the expression of other allergens, at least based on the varieties examined. In other words, the allergens appear to vary in their expression in response to one or more variables independently of one another. For example, when KTI 1 and KTI 3 were examined as a class of related proteins (i.e., trypsin inhibitors) they were shown to not vary as a related pair of proteins across the three soybean varieties. MANOVA analysis (Supplementary Table 3) showed highly significant variety-by-allergen interaction indicating that an increase in one of these trypsin inhibitors is not accompanied by a similar increase in the other; location by allergen interaction was also significant, though not as strong as the variety by allergen interaction. A more graphical explanation of the effect shows that when KTI 1 concentrations were plotted against KTI 3 (**Figure 5**) varieties A and B1 are similar, but concentrations of KTI 1 for these two varieties are substantially higher in comparison to B2. Further analysis by ANOVA on the summed concentrations of KTI 1 and KTI 3 demonstrated that the sum differs significantly by variety (*F* = 51.71, df = 2,16, *p* < .0001) though not by location (*F* = 1.88, df = 8,16, *p* = 0.136); again, another reflection of the impact of soybean variety on allergen concentration. This degree of independence for KTI 1 and KTI 3 was also observed by Stevenson et al. (2012) in the way in which these two allergens and their expression were impacted by genotype and location (2012).

An interesting exception to the general lack of correlation among the allergens by variety or location was a comparison of β-conglycinin α-subunit to the total glycinin content. The ratio of β-conglycinin α-subunit to total glycinin concentration was calculated for each sample, and ANOVA was carried out on the ratios to test whether the ratio differed by variety or location. The main effect for variety was highly significant and indicated that the concentration of β-conglycinin α-subunit relative to glycinin was dependent on the variety of soybean (bottom of **Table 1**). This may not be surprising since β-conglycinin α-subunit and glycinin are storage proteins and the abundance of one may influence the content of the other in terms of overall seed storage protein levels and their potential biological limitations for expression in the seed. This has been observed before in early attempts to quantify the amounts of these two important proteins in soybeans which were based on studies of different varieties grown at the same location (Boyer, 1982).

## Discussion

Environmental factors and genetic traits have long been known to influence quality and productivity of agricultural crops (Harada et al., 1983). Environmental factors encompass both biotic and abiotic elements that influence a plant’s growth and development. Biotic factors affecting agricultural production include, but are not limited to, pests, parasites, weeds, and predators. Abiotic factors include temperature, rainfall, irrigation, and pesticide and herbicide application. While many studies have addressed the correlation between genetic and environmental factors affecting crop productivity and composition there are fewer studies that have addressed the impact of these factors on specific protein expression. A recent investigation on allergen variability within four conventional soybean varieties harvested from Georgia, Iowa, Kansas, Nebraska, Ontario, and Pennsylvania during seasonal growth conditions in 2007 revealed diverse environmental growth conditions (spanning three agricultural climate zones) had a greater influence on allergen variability than genetic background (Stevenson et al., 2012). However, due to the single sampling from each location, it was unclear whether such environmental variation was also possible within a limited geographical region.

In the current study, we investigated the effect of environment and soybean variety on the levels of the same soybean seed allergens from field productions of three commercial varieties in nine Midwestern locations (seven in IA, and one each in IL and IN) within a single climate zone. Soybean plots were planted within the typical United States soybean growing region during the growing season of 2009. All plots received conventional herbicide treatment with commercially available herbicides in accordance with typical agronomic practice. The results presented here clearly demonstrate that both genetic and environmental factors influence protein allergen content in soybean seed. It was clear that in the case of the KTI proteins the KTI 3 protein had a much greater range in abundance for two of the varieties (B1 and B2) compared to the variety A (**Figure 5**). Glycinin and β-conglycinin α-subunit abundances were affected by both soybean variety and by growing location. This is important since they are not only major soybean allergens, but they are major seed storage proteins accounting for over 30% of total protein. The four subunits of glycinin displayed differences in seed concentrations with glycinin G1 and glycinin G2 being most abundant followed by glycinin G4 and glycinin G3, and this is consistent with previous studies (Houston et al., 2011; Stevenson et al., 2012) in terms of ranking these isoforms by their abundance. Glycinin G1 and glycinin G2 also display similar patterns of accumulation as influenced by the variety, but were less influenced by growing location (i.e., environmental influence).

To analyze how the concentration of one allergen coincided with the concentration of another, the ratio of β-conglycinin α-subunit was compared to the sum of the four glycinin isoforms and the conclusion reached was that variety does impact the relationship between β-conglycinin α-subunit and total glycinin. Earlier studies have demonstrated that glycinin content can indeed vary by soybean variety and there is evidence that at least 20% of Japanese soybean varieties lack the group IIa A_5_A_4_B_3_ glycinin subunit (Harada et al., 1983; Hughes and Murphy, 1983), which would be expected to impact a quantitative measure of the glycinin subunit(s). Environmental growth conditions are also known to significantly impact the levels of glycinin and β-conglycinin levels in mature soybean seeds (Helm et al., 1998; Fehr et al., 2003; Stevenson et al., 2012). Murphy and Resurreccion (1984) reported that environmental differences were more influential than varietal differences in the abundances of glycinin and β-conglycinin of four cultivars (i.e., varieties) grown in different years at different locations. Although the different locations were within the state of IA, there appeared to be a greater magnitude of differences among sites compared to the small differences observed when two varieties were grown side-by-side under the same conditions within the same year. The conclusions that Murphy and Resurreccion (1984) draw are different than those observed herein and are likely a result of a combination of factors; (a) much different soybean genetic backgrounds and (b) relatively coarse detection methods used which they acknowledge can have a differential effect on measured glycinin content as a proportion of total protein. In particular, there was relatively little variation (3.3–10.5%) which may have been outside the range of precision given the unknown level of variation in the total protein extraction method that was common at the time. In other work, Barros et al. (2010), showed with non-specific protein profiling that site location can be a dominant influence when protein profiling across maize varieties grown during a single season even when the distance between growing locations is relatively short at ∼140 miles (∼225 km); this was apart from the fact that variety did not appear to be an important variable correlated with expression. In contrast, our study indicates that although environmental factors could be detected statistically, the variety of soybean was more dominant in explaining the observed variation in the concentrations of the glycinins and β-conglycinin α-subunit. As an example, the β-conglycinin α-subunit expression was strongly influenced by the variety with almost a twofold lower concentration in the variety B1 compared to the other two varieties (Supplementary Table 4). The overall dominance of the variety effect on allergen content in this study may be due to the fact that seven of the nine growing locations were relatively close to one another and not necessarily representative of the overall variation in United States soybean growing regions. Indeed, studies carried out by Stevenson et al. (2012) demonstrated that growth locations that are geographically distinct exert greater influence than varietal differences on allergen expression.

## Conclusion

These data employ a new quantitative assay for the determination of a select set of biologically important proteins in a complex matrix. A quantitative approach lessens the impact of assay-specific, sample-to-sample variation while inherently increasing the ability to discriminate study design-specific variables (i.e., better precision). We examined two of the most important variables that affect protein abundance in a complex soybean seed matrix, genetic (variety) and environmental influences. These hypothesized influential variables on allergen expression are characterized as general variables as we do not know the tangible variances of the multiple genes that may vary within a cultivar. With regard to environmental variables, the site locations for the field trial from which the samples in this study were taken are within one growing region for soybean, though there is an expectation that site-to-site variances in actual environmental conditions such as weather, soil, etc., are important agronomically. However, specific measures of both genetics (detailed gene descriptions) and abiotic/biotic environmental conditions were beyond the scope of the work herein in terms of a statistical correlation approach.

We identified that as few as three different soybean varieties can display statistically detectable differential accumulation of protein allergens. Allergens were the focus because analytical detection of these proteins could lead to a better understanding of how best to interpret potential changes in their accumulation. The expectation is that a quantitative assessment of these allergens as well as any other proteins by the methods employed here can be used for the purpose of investigating plant protein expression from a targeted proteomic perspective. Although commodity soybeans are blended to a high degree and the natural variation in expression would be tempered, the ability to measure allergens will likely provide an important analytical technique to support clinical studies of soybean allergy. The concept of natural variability will also play a role in understanding regulatory questions regarding soybean allergens, as a recent review has highlighted (Ladics et al., 2014). A multiplex approach, especially one that is reliably quantitative, can support a greater degree of precision in the design of future studies that seek to understand allergen threshold exposure and related aspects of clinical food allergy. Such a quantitative approach represents a major improvement over serum-based assays by reducing the inconsistency and variability associated with such studies. In future surveys of the potential impact of genetics and environment on allergen expression, it can be imagined that extensive specific field trial designs could catalog and measure many of the discrete variables. This would allow much more extensive means comparisons and principle component type analyses to better understand more than the very top-level variables of “genetics” and “environment” we have assessed in this work. In the near term, this work supports a better understanding of the broad sources of variation with regard to natural protein allergen expression that we consider a stepping stone to providing quantitative allergen detection methodology.

## Author Contributions

JT and SS sponsored and performed all the laboratory analyses and data compilation. CB provided the statistical analyses. CH-G supported for the sample collection and coordination. CH-G, RH, GL, LP, JW, ND, and SM provided the study design guidance and manuscript editing. SM provided the primary manuscript text, tables, and figures and the editing coordination.

## Disclaimer

The views and conclusions expressed in this article are those of the authors and do not necessarily represent the policies or positions of their organizations.

## Conflict of Interest Statement

Some authors are employed by organizations that develop and market transgenic seed.

## Footnotes

1 http://comparedatabase.org

2 www.sas.com
