# Supplementary material for: Variation in Seed Allergen Content From Three Varieties of Soybean Cultivated in Nine Different Locations in Iowa, Illinois, and Indiana
Source: Front Plant Sci. 2018 Jul 23;9:1025. doi: 10.3389/fpls.2018.01025 (PMC6065051; doi:10.3389/fpls.2018.01025)
Supplement: Supplementary file 1 [file Table_1.docx]

Supplementary Material

Variation in Seed Allergen Content from Three Varieties of Soybean Cultivated in Nine Different Locations in Iowa, Illinois, and Indiana

Scott McClain1*, Severin E. Stevenson2, Cavell Brownie3 Corinne Herouet-Guicheney4, Rod A. Herman5, Gregory S. Ladics6, Laura Privalle7, Jason M. Ward8, Nancy Doerrer9, Jay J. Thelen10

*** Correspondence:** Scott McClain: scottmcclain24@gmail.com

**Supplementary Table 1.** Target protein and peptide information for quantifying each respective protein allergen using mass spectrometry. (fmol = femtomole; g/mol is molar mass of peptide; inj = injection).

| **Target Name** | **Glyma #  (for protein target)** | **Allergen Symbol** | **Peptide Sequence** | **fmol/inj (1ug)** | **g/mol** |
| --- | --- | --- | --- | --- | --- |
| Glycinin G1 | Glyma03g32030.1 | GlyG1 | VLIVPQNFVVAAR | 200 | 1425.9 |
| Glycinin G2 | Glyma03g32020.1 Glyma03g32020.2 | GlyG2 | NLQGENEEEDSGAIVTVK | 100 | 1931.9 |
| Glycinin G3 | Glyma19g34780.1 | GlyG3 | FYLAGNQEQEFLQYQPQK | 100 | 2231.1 |
| Glycinin G4 | Glyma10g04280.1 | GlyG4 | VESEGGLIQTWNSQHPELK | 100 | 2152.1 |
| Beta-conglycinin, Alpha subunit | Glyma20g28650.1 Glyma20g28650.2 Glyma20g28660.1 | BconA1 | LITLAIPVNKPGR | 100 | 1391.9 |
| Kunitz trypsin inhibitor 1 | Glyma01g10900.1 | KTI1 | DTVDGWFNIER | 100 | 1351.6 |
| Kunitz trypsin inhibitor 3 | Glyma08g45530.1 | KTI3 | FIAEGHPLSLK | 100 | 1211.7 |
| Gly m Bd 28k | Glyma11g15870.1 | AllGly28 | DGPLEFFGFSTSAR | 100 | 1530.7 |
| 34 kDa maturing seed protein | Glyma08g12270.1 | P34 | EESETLVSAR | 1 | 1120.5 |
