# Supplementary material for: Variation in Seed Allergen Content From Three Varieties of Soybean Cultivated in Nine Different Locations in Iowa, Illinois, and Indiana
Source: Front Plant Sci. 2018 Jul 23;9:1025. doi: 10.3389/fpls.2018.01025 (PMC6065051; doi:10.3389/fpls.2018.01025)
Supplement: Supplementary file 2 [file Table_2.docx]

Supplementary Material

Variation in Seed Allergen Content from Three Varieties of Soybean Cultivated in Nine Different Locations in Iowa, Illinois, and Indiana

Scott McClain1*, Severin E. Stevenson2, Cavell Brownie3 Corinne Herouet-Guicheney4, Rod A. Herman5, Gregory S. Ladics6, Laura Privalle7, Jason M. Ward8, Nancy Doerrer9, Jay J. Thelen10

*** Correspondence:** Scott McClain: scottmcclain24@gmail.com

**Supplementary Table 2.** Mass spectrometry assay parameters.

| **Peptide** | **Peptide Name** | **Precursor (Th)** | **Precursor Charge** | **Product (Th)** | **Product Charge** | **Ion Type** |
| --- | --- | --- | --- | --- | --- | --- |
| EESETLVSAR | P34 | 560.778 | 2 | 432.256 | 1 | y4 |
| EESETLVSAR | P34 | 560.778 | 2 | 545.34 | 1 | y5 |
| EESETLVSAR | P34 | 560.778 | 2 | 646.388 | 1 | y6 |
| EESETLVSAR | P34 | 560.778 | 2 | 862.462 | 1 | y8 |
| EESETLVSAR | P34 | 560.778 | 2 | 991.505 | 1 | y9 |
| EESETLVSAR | P34 | 560.778 | 2 | 333.188 | 1 | y3 |
| EESETLVSAR[HeavyR] | P34 | 565.782 | 2 | 442.264 | 1 | y4 |
| EESETLVSAR[HeavyR] | P34 | 565.782 | 2 | 555.348 | 1 | y5 |
| EESETLVSAR[HeavyR] | P34 | 565.782 | 2 | 656.396 | 1 | y6 |
| EESETLVSAR[HeavyR] | P34 | 565.782 | 2 | 872.471 | 1 | y8 |
| EESETLVSAR[HeavyR] | P34 | 565.782 | 2 | 1001.513 | 1 | y9 |
| EESETLVSAR[HeavyR] | P34 | 565.782 | 2 | 343.196 | 1 | y3 |
| NLQGENEEEDSGAIVTVK | GlyG2 | 966.463 | 2 | 1390.669 | 1 | y13 |
| NLQGENEEEDSGAIVTVK | GlyG2 | 966.463 | 2 | 1276.626 | 1 | y12 |
| NLQGENEEEDSGAIVTVK | GlyG2 | 966.463 | 2 | 1147.584 | 1 | y11 |
| NLQGENEEEDSGAIVTVK | GlyG2 | 966.463 | 2 | 1018.541 | 1 | y10 |
| NLQGENEEEDSGAIVTVK | GlyG2 | 966.463 | 2 | 889.498 | 1 | y9 |
| NLQGENEEEDSGAIVTVK[HeavyK] | GlyG2 | 970.47 | 2 | 1398.683 | 1 | y13 |
| NLQGENEEEDSGAIVTVK[HeavyK] | GlyG2 | 970.47 | 2 | 1284.64 | 1 | y12 |
| NLQGENEEEDSGAIVTVK[HeavyK] | GlyG2 | 970.47 | 2 | 1155.598 | 1 | y11 |
| NLQGENEEEDSGAIVTVK[HeavyK] | GlyG2 | 970.47 | 2 | 1026.555 | 1 | y10 |
| NLQGENEEEDSGAIVTVK[HeavyK] | GlyG2 | 970.47 | 2 | 897.513 | 1 | y9 |
| FIAEGHPLSLK | KTI3 | 404.564 | 3 | 460.312 | 1 | y4 |
| FIAEGHPLSLK | KTI3 | 404.564 | 3 | 557.365 | 1 | y5 |
| FIAEGHPLSLK | KTI3 | 404.564 | 3 | 694.424 | 1 | y6 |
| FIAEGHPLSLK | KTI3 | 404.564 | 3 | 751.446 | 1 | y7 |
| FIAEGHPLSLK | KTI3 | 404.564 | 3 | 880.488 | 1 | y8 |
| FIAEGHPLSLK | KTI3 | 404.564 | 3 | 951.525 | 1 | y9 |
| FIAEGHPLSLK[HeavyK] | KTI3 | 407.236 | 3 | 468.327 | 1 | y4 |
| FIAEGHPLSLK[HeavyK] | KTI3 | 407.236 | 3 | 565.379 | 1 | y5 |
| FIAEGHPLSLK[HeavyK] | KTI3 | 407.236 | 3 | 702.438 | 1 | y6 |
| FIAEGHPLSLK[HeavyK] | KTI3 | 407.236 | 3 | 759.46 | 1 | y7 |
| FIAEGHPLSLK[HeavyK] | KTI3 | 407.236 | 3 | 888.502 | 1 | y8 |
| FIAEGHPLSLK[HeavyK] | KTI3 | 407.236 | 3 | 959.539 | 1 | y9 |
| VESEGGLIQTWNSQHPELK | GlyG4 | 718.027 | 3 | 854.444 | 2 | y15 |
| VESEGGLIQTWNSQHPELK | GlyG4 | 718.027 | 3 | 962.481 | 2 | y17 |
| VESEGGLIQTWNSQHPELK | GlyG4 | 718.027 | 3 | 486.292 | 1 | y4 |
| VESEGGLIQTWNSQHPELK | GlyG4 | 718.027 | 3 | 1367.67 | 1 | y11 |
| VESEGGLIQTWNSQHPELK | GlyG4 | 718.027 | 3 | 1239.611 | 1 | y10 |
| VESEGGLIQTWNSQHPELK | GlyG4 | 718.027 | 3 | 1138.563 | 1 | y9 |
| VESEGGLIQTWNSQHPELK | GlyG4 | 718.027 | 3 | 740.881 | 2 | y12 |
| VESEGGLIQTWNSQHPELK | GlyG4 | 718.027 | 3 | 445.192 | 1 | b3 |
| VESEGGLIQTWNSQHPELK[HeavyK] | GlyG4 | 720.698 | 3 | 858.451 | 2 | y15 |
| VESEGGLIQTWNSQHPELK[HeavyK] | GlyG4 | 720.698 | 3 | 966.488 | 2 | y17 |
| VESEGGLIQTWNSQHPELK[HeavyK] | GlyG4 | 720.698 | 3 | 494.306 | 1 | y4 |
| VESEGGLIQTWNSQHPELK[HeavyK] | GlyG4 | 720.698 | 3 | 1375.684 | 1 | y11 |
| VESEGGLIQTWNSQHPELK[HeavyK] | GlyG4 | 720.698 | 3 | 1247.625 | 1 | y10 |
| VESEGGLIQTWNSQHPELK[HeavyK] | GlyG4 | 720.698 | 3 | 1146.578 | 1 | y9 |
| VESEGGLIQTWNSQHPELK[HeavyK] | GlyG4 | 720.698 | 3 | 744.888 | 2 | y12 |
| VESEGGLIQTWNSQHPELK[HeavyK] | GlyG4 | 720.698 | 3 | 445.192 | 1 | b3 |
| LITLAIPVNKPGR | BconA | 464.629 | 3 | 457.288 | 1 | y4 |
| LITLAIPVNKPGR | BconA | 464.629 | 3 | 571.331 | 1 | y5 |
| LITLAIPVNKPGR | BconA | 464.629 | 3 | 670.399 | 1 | y6 |
| LITLAIPVNKPGR | BconA | 464.629 | 3 | 767.452 | 1 | y7 |
| LITLAIPVNKPGR | BconA | 464.629 | 3 | 880.536 | 1 | y8 |
| LITLAIPVNKPGR | BconA | 464.629 | 3 | 951.573 | 1 | y9 |
| LITLAIPVNKPGR | BconA | 464.629 | 3 | 1064.657 | 1 | y10 |
| LITLAIPVNKPGR | BconA | 464.629 | 3 | 1165.705 | 1 | y11 |
| LITLAIPVNKPGR[HeavyR] | BconA | 467.965 | 3 | 467.296 | 1 | y4 |
| LITLAIPVNKPGR[HeavyR] | BconA | 467.965 | 3 | 581.339 | 1 | y5 |
| LITLAIPVNKPGR[HeavyR] | BconA | 467.965 | 3 | 680.407 | 1 | y6 |
| LITLAIPVNKPGR[HeavyR] | BconA | 467.965 | 3 | 777.46 | 1 | y7 |
| LITLAIPVNKPGR[HeavyR] | BconA | 467.965 | 3 | 890.544 | 1 | y8 |
| LITLAIPVNKPGR[HeavyR] | BconA | 467.965 | 3 | 961.581 | 1 | y9 |
| LITLAIPVNKPGR[HeavyR] | BconA | 467.965 | 3 | 1074.665 | 1 | y10 |
| LITLAIPVNKPGR[HeavyR] | BconA | 467.965 | 3 | 1175.713 | 1 | y11 |
| FYLAGNQEQEFLQYQPQK | GlyG3 | 1116.042 | 2 | 663.346 | 1 | y5 |
| FYLAGNQEQEFLQYQPQK | GlyG3 | 1116.042 | 2 | 791.404 | 1 | y6 |
| FYLAGNQEQEFLQYQPQK | GlyG3 | 1116.042 | 2 | 904.488 | 1 | y7 |
| FYLAGNQEQEFLQYQPQK | GlyG3 | 1116.042 | 2 | 1051.557 | 1 | y8 |
| FYLAGNQEQEFLQYQPQK | GlyG3 | 1116.042 | 2 | 1180.599 | 1 | y9 |
| FYLAGNQEQEFLQYQPQK | GlyG3 | 1116.042 | 2 | 372.224 | 1 | y3 |
| FYLAGNQEQEFLQYQPQK[HeavyK] | GlyG3 | 1120.049 | 2 | 671.36 | 1 | y5 |
| FYLAGNQEQEFLQYQPQK[HeavyK] | GlyG3 | 1120.049 | 2 | 799.418 | 1 | y6 |
| FYLAGNQEQEFLQYQPQK[HeavyK] | GlyG3 | 1120.049 | 2 | 912.502 | 1 | y7 |
| FYLAGNQEQEFLQYQPQK[HeavyK] | GlyG3 | 1120.049 | 2 | 1059.571 | 1 | y8 |
| FYLAGNQEQEFLQYQPQK[HeavyK] | GlyG3 | 1120.049 | 2 | 1188.613 | 1 | y9 |
| FYLAGNQEQEFLQYQPQK[HeavyK] | GlyG3 | 1120.049 | 2 | 380.238 | 1 | y3 |
| VLIVPQNFVVAAR | GlyG1 | 713.432 | 2 | 425.312 | 1 | b3 |
| VLIVPQNFVVAAR | GlyG1 | 713.432 | 2 | 501.28 | 2 | y9 |
| VLIVPQNFVVAAR | GlyG1 | 713.432 | 2 | 1001.552 | 1 | y9 |
| VLIVPQNFVVAAR | GlyG1 | 713.432 | 2 | 326.243 | 1 | b2 |
| VLIVPQNFVVAAR | GlyG1 | 713.432 | 2 | 904.499 | 1 | y8 |
| VLIVPQNFVVAAR | GlyG1 | 713.432 | 2 | 1100.621 | 1 | y10 |
| VLIVPQNFVVAAR | GlyG1 | 713.432 | 2 | 776.441 | 1 | y7 |
| VLIVPQNFVVAAR[HeavyR] | GlyG1 | 718.437 | 2 | 425.312 | 1 | b3 |
| VLIVPQNFVVAAR[HeavyR] | GlyG1 | 718.437 | 2 | 506.284 | 2 | y9 |
| VLIVPQNFVVAAR[HeavyR] | GlyG1 | 718.437 | 2 | 1011.56 | 1 | y9 |
| VLIVPQNFVVAAR[HeavyR] | GlyG1 | 718.437 | 2 | 326.243 | 1 | b2 |
| VLIVPQNFVVAAR[HeavyR] | GlyG1 | 718.437 | 2 | 914.508 | 1 | y8 |
| VLIVPQNFVVAAR[HeavyR] | GlyG1 | 718.437 | 2 | 1110.629 | 1 | y10 |
| VLIVPQNFVVAAR[HeavyR] | GlyG1 | 718.437 | 2 | 786.449 | 1 | y7 |
| DTVDGWFNIER | KTI1 | 676.317 | 2 | 531.288 | 1 | y4 |
| DTVDGWFNIER | KTI1 | 676.317 | 2 | 921.457 | 1 | y7 |
| DTVDGWFNIER | KTI1 | 676.317 | 2 | 678.356 | 1 | y5 |
| DTVDGWFNIER | KTI1 | 676.317 | 2 | 1036.484 | 1 | y8 |
| DTVDGWFNIER | KTI1 | 676.317 | 2 | 864.436 | 1 | y6 |
| DTVDGWFNIER[HeavyR] | KTI1 | 681.322 | 2 | 541.296 | 1 | y4 |
| DTVDGWFNIER[HeavyR] | KTI1 | 681.322 | 2 | 931.466 | 1 | y7 |
| DTVDGWFNIER[HeavyR] | KTI1 | 681.322 | 2 | 688.365 | 1 | y5 |
| DTVDGWFNIER[HeavyR] | KTI1 | 681.322 | 2 | 1046.492 | 1 | y8 |
| DTVDGWFNIER[HeavyR] | KTI1 | 681.322 | 2 | 874.444 | 1 | y6 |
| DGPLEFFGFSTSAR | AllGly28 | 765.865 | 2 | 1148.536 | 1 | y10 |
| DGPLEFFGFSTSAR | AllGly28 | 765.865 | 2 | 1019.494 | 1 | y9 |
| DGPLEFFGFSTSAR | AllGly28 | 765.865 | 2 | 872.426 | 1 | y8 |
| DGPLEFFGFSTSAR | AllGly28 | 765.865 | 2 | 725.357 | 1 | y7 |
| DGPLEFFGFSTSAR | AllGly28 | 765.865 | 2 | 1415.695 | 1 | y13 |
| DGPLEFFGFSTSAR | AllGly28 | 765.865 | 2 | 1358.673 | 1 | y12 |
| DGPLEFFGFSTSAR | AllGly28 | 765.865 | 2 | 1261.621 | 1 | y11 |
| DGPLEFFGFSTSAR[HeavyR] | AllGly28 | 770.869 | 2 | 1158.545 | 1 | y10 |
| DGPLEFFGFSTSAR[HeavyR] | AllGly28 | 770.869 | 2 | 1029.502 | 1 | y9 |
| DGPLEFFGFSTSAR[HeavyR] | AllGly28 | 770.869 | 2 | 882.434 | 1 | y8 |
| DGPLEFFGFSTSAR[HeavyR] | AllGly28 | 770.869 | 2 | 735.365 | 1 | y7 |
| DGPLEFFGFSTSAR[HeavyR] | AllGly28 | 770.869 | 2 | 1425.703 | 1 | y13 |
| DGPLEFFGFSTSAR[HeavyR] | AllGly28 | 770.869 | 2 | 1368.682 | 1 | y12 |
| DGPLEFFGFSTSAR[HeavyR] | AllGly28 | 770.869 | 2 | 1271.629 | 1 | y11 |
