# Supplementary material for: Variation in Seed Allergen Content From Three Varieties of Soybean Cultivated in Nine Different Locations in Iowa, Illinois, and Indiana
Source: Front Plant Sci. 2018 Jul 23;9:1025. doi: 10.3389/fpls.2018.01025 (PMC6065051; doi:10.3389/fpls.2018.01025)
Supplement: Supplementary file 3 [file Table_3.docx]

Supplementary Material

Variation in Seed Allergen Content from Three Varieties of Soybean Cultivated in Nine Different Locations in Iowa, Illinois, and Indiana

Scott McClain1*, Severin E. Stevenson2, Cavell Brownie3 Corinne Herouet-Guicheney4, Rod A. Herman5, Gregory S. Ladics6, Laura Privalle7, Jason M. Ward8, Nancy Doerrer9, Jay J. Thelen10

*** Correspondence:** Scott McClain: scottmcclain24@gmail.com

**Supplementary Table 3.** MANOVA results for pairs of the most abundant allergens. Glycinin sub-units were summed to create “total glycinin” values for analysis. KTI1 and KTI3 were similarly summed to create “Total KTI”. Beta-conglycinin is the same as “β-conglycinin α-subunit” listed elsewhere. Variety is equivalent to the descriptor “genotype”.

| **Beta-conglycinin and total glycinin** | |  |  |  |
| --- | --- | --- | --- | --- |
| **Model Effect** | **DF** | **Den, DF** | **F value** | ***p*-value** |
| Genotype | 2 | 54 | 291.07 | <0.0001 |
| Location | 8 | 54 | 3.65 | 0.0018 |
| Location*Genotype | 16 | 54 | 2.53 | 0.0057 |
| Allergengrp | 1 | 54 | 189838 | <0.0001 |
| Genotype*allergengrp | 2 | 54 | 1739.29 | <0.0001 |
| Location*allergengrp | 8 | 54 | 8.59 | <0.0001 |
| Location*Genotype*allergen | 16 | 54 | 2.99 | 0.0013 |
|  |  |  |  |  |
| **Beta-conglycinin and total KTI** | |  |  |  |
| **Model Effect** | **DF** | **Den, DF** | **F value** | ***p*-value** |
| Genotype | 2 | 54 | 176.21 | <0.0001 |
| Location | 8 | 54 | 4.04 | 0.0008 |
| Location*Genotype | 16 | 54 | 3.74 | 0.0001 |
| Allergengrp | 1 | 54 | 33313.7 | <0.0001 |
| Genotype*allergengrp | 2 | 54 | 2621.23 | <0.0001 |
| Location*allergengrp | 8 | 54 | 20.73 | <0.0001 |
| Location*Genotype*allergen | 16 | 54 | 7.47 | <0.0001 |
|  |  |  |  |  |
| **Total glycinin and total KTI** | |  |  |  |
| **Model Effect** | **DF** | **Den, DF** | **F value** | ***p*-value** |
| Genotype | 2 | 54 | 84.31 | <0.0001 |
| Location | 8 | 54 | 2.73 | 0.0133 |
| Location*Genotype | 16 | 54 | 3.66 | 0.0002 |
| Allergengrp | 1 | 54 | 1112747 | <0.0001 |
| Genotype*allergengrp | 2 | 54 | 2544.47 | <0.0001 |
| Location*allergengrp | 8 | 54 | 78.36 | <0.0001 |
| Location*Genotype*allergen | 16 | 54 | 29.61 | <0.0001 |
|  |  |  |  |  |
| **KTI1 and KTI3** |  |  |  |  |
| **Model Effect** | **DF** | **Den, DF** | **F value** | ***p*-value** |
| Genotype | 2 | 54 | 946.27 | <0.0001 |
| Location | 8 | 54 | 14.34 | <0.0001 |
| Location*Genotype | 16 | 54 | 8.28 | <0.0001 |
| Protein_name | 1 | 54 | 10159.9 | <0.0001 |
| Genotype*Protein_name | 2 | 54 | 4555.33 | <0.0001 |
| Location*Protein_name | 8 | 54 | 10.15 | <0.0001 |
| Location*Genotype*Protein | 16 | 54 | 4.93 | <0.0001 |
