# Supplementary material for: Variation in Seed Allergen Content From Three Varieties of Soybean Cultivated in Nine Different Locations in Iowa, Illinois, and Indiana
Source: Front Plant Sci. 2018 Jul 23;9:1025. doi: 10.3389/fpls.2018.01025 (PMC6065051; doi:10.3389/fpls.2018.01025)
Supplement: Supplementary file 4 [file Table_4.docx]

Supplementary Material

Variation in Seed Allergen Content from Three Varieties of Soybean Cultivated in Nine Different Locations in Iowa, Illinois, and Indiana

Scott McClain1*, Severin E. Stevenson2, Cavell Brownie3 Corinne Herouet-Guicheney4, Rod A. Herman5, Gregory S. Ladics6, Laura Privalle7, Jason M. Ward8, Nancy Doerrer9, Jay J. Thelen10

*** Correspondence:** Scott McClain: scottmcclain24@gmail.com

**Supplementary Table 4.** Mean concentrations (μg/mg total protein) by variety for each allergen.

| **Allergen protein** | **Variety A** | **Variety B1** | **Variety B2** |
| --- | --- | --- | --- |
| P34 (Gly m Bd 30k) | 0.032 | 0.036 | 0.024 |
| β-conglycinin  α-subunit | 19.18 | 11.08 | 20.57 |
| Gly m Bd 28k | 1.83 | 1.71 | 1.83 |
| Glycinin G1 | 66.4 | 78.15 | 81.54 |
| Glycinin G2 | 59.06 | 65.38 | 68.54 |
| Glycinin G3 | 3.21 | 4.09 | 4.13 |
| Glycinin G4 | 6.81 | 7.25 | 7.14 |
| KTI1 | 3.24 | 3.03 | 1.24 |
| KTI3 | 3.54 | 3.77 | 3.6 |
