# Supplementary material for: Variation in Seed Allergen Content From Three Varieties of Soybean Cultivated in Nine Different Locations in Iowa, Illinois, and Indiana
Source: Front Plant Sci. 2018 Jul 23;9:1025. doi: 10.3389/fpls.2018.01025 (PMC6065051; doi:10.3389/fpls.2018.01025)
Supplement: Supplementary file 5 [file Table_5.docx]

Supplementary Material

Variation in Seed Allergen Content from Three Varieties of Soybean Cultivated in Nine Different Locations in Iowa, Illinois, and Indiana

Scott McClain1*, Severin E. Stevenson2, Cavell Brownie3 Corinne Herouet-Guicheney4, Rod A. Herman5, Gregory S. Ladics6, Laura Privalle7, Jason M. Ward8, Nancy Doerrer9, Jay J. Thelen10

*** Correspondence:** Scott McClain: scottmcclain24@gmail.com

**Supplementary Table 5.** Site-Specific Soil Description and Weather Conditions Associated with the 2009 Grain Production Trials.

| **Closest Town** | **GPS Coordinates** | **Soil Type** | **Soil pH** | **Organic Matter**  **(%)** | **Month** | **Avg Max Temperature**  **(°F)** | **Av. Min Temperature**  **(°F)** | **Total Rainfall**  **(Inch)** |
| --- | --- | --- | --- | --- | --- | --- | --- | --- |
| Adel, Iowa | 41.70291  -94.16245 | Caniesteo Silty Clay | 7.6 – 8.4 | 0.1 – 7.0 | May  June  July  August  September  October | 77  81  80  79  77  53 | 56  61  60  61  54  38 | 2.95  5.20  3.10  4.79  0.88  7.15 |
| Glidden, Iowa | 42048600  -94.601260 | Nicollet Loam, Webster Clay Loam | 6.7 – 7.0 | 5.3 – 6.3 | May  June  July  August  September  October | 74  81  80  79  78  54 | 54  61  60  60  51  39 | 1.89  4.81  3.63  4.66  0.45  4.22 |
| Iowa Falls, Iowa | 42.559240  -93.470050 | Webster Silty Clay Loam, Clarion Loam, Caniesteo Silty Clay Loam | 6.9 – 7.9 | 1.69 – 4.9 | May  June  July  August  September  October | 65  75  76  74  73  50 | 46  57  56  56  48  36 | 2.34  3.65  2.07  0.94  2.86  4.64 |
| Marcus, Iowa | 42.877536  -95.819992 | Primghar Silty Clay Loam | 6.5 | 5.5 | May  June  July  August  September  October | 73  78  79  79  76  51 | 49  59  59  59  53  37 | 0.82  0.83  0.65  1.16  1.11  4.53 |
| Mediapolis, Iowa | 41.020920  -91.141540 | Taintor Silty Clay Loam | 6.4 | 2.35 | May  June  July  August  September  October | 77  83  80  82  77  57 | 55  62  58  60  51  39 | 4.76  7.32  4.60  9.47  1.01  7.14 |
| Perry, Iowa | 41.792190  -94.067280 | Clarion Loam, Caniesteo Silty Clay Loam | 5.6 – 7.8 | 1.43 – 3.37 | May  June  July  August  September  October | 74  80  80  79  77  53 | 53  61  59  60  51  37 | 2.95  5.20  3.10  4.79  0.88  7.15 |
| Winterset, Iowa | 41.032420  -94.095100 | Sharpsburg Silty Clay | 6.0 | 1.76 | May  June  July  August  September  October | 76  84  84  83  82  56 | 51  59  57  58  49  35 | 3.88  6.84  2.97  5.38  1.42  6.25 |
| Fithian, Illinois | 40.177133  -87.838050 | Drummer Silty Clay Loam | 6.1 | 4.5 - 7.0 | May  June  July  August  September  October | 80  82  78  80  76  58 | 59  63  61  63  57  43 | 5.90  3.95  4.01  5.20  1.93  7.75 |
| Sharpsville, Indiana | 40.374250  -86.215917 | Patton Silty Clay Loam | 6.7 | 4.5 | May  June  July  August  September  October  November | 86  86  85  85  79  60  59 | 55  61  58  58  54  41  30 | 4.87  3.70  3.36  2.94  1.53  5.84  1.52 |
